# Purification and Optimization of Extracellular Lipase from a Novel Strain *Kocuria flava* Y4

**DOI:** 10.1155/2022/6403090

**Published:** 2022-02-05

**Authors:** Adyasa Barik, Sudip Kumar Sen, Geetanjali Rajhans, Sangeeta Raut

**Affiliations:** ^1^Centre for Biotecnology, School of Pharmaceutical Sciences, Siksha ‘O' Anusandhan (Deemed to be University), Bhubaneswar 751003, Odisha, India; ^2^Biostadt India Limited, Waluj, Aurangabad, Maharashtra 431136, India

## Abstract

The exogenous lipolytic activities of *Kocuria* sp. have been recognized earlier but the genus further contains many more unexplored strains. In this study, the extracellular lipase activity of *Kocuria flava* Y4 (GenBank accession no. MT773277), isolated from *Dioscorea villosa* during our previous study, was regulated by different physicochemical parameters, such as pH, temperature, shaking speed, and incubation time. For efficient immobilization of the extracellular lipase, 4% sodium alginate, 50 mL of 25 nM CaCl_2_.2H_2_O solution, and 15 min. Hardening time of gel beads in calcium chloride was used. For the first time, *K*. *flava* Y4 lipase was purified using ammonium sulphate precipitation followed by dialysis and DEAE-Sepharose anion exchange chromatography with Sepharose-6B gel filtration chromatography, yielding ∼15-fold purified lipase with a final yield of 96 U/mL. The SDS-PAGE of purified lipase displayed a single strong band, indicating a monomeric protein of 45 kDa. At a temperature of 35°C and pH 8, the purified lipase showed maximum hydrolytic activity. Using p-nitrophenyl acetate (p-NPA) as the hydrolysis substrate, the values of *K*_*m*_ and *V*_max_ derived from the Lineweaver–Burk plot were 4.625 mM and 125 mol/min^−1^mg^−1^, respectively.

## 1. Introduction

The dawn of enzymology in biotechnology industry as an important breakthrough brings a global enzyme usage of approximately $1.5 billion in 2000 [1]. Hydrolytic enzymes like amylases, proteases, esterases, lipases, and amidases currently dominate the worldwide market of industrial enzymes. Lipases (glycerol ester hydrolase; EC 3.1.1.3) are carboxyl esterases involved in the catalysis of carboxylic esters hydrolysis in aqueous media as well as the production of these esters in water-restricted media, showing excellent regio-, chemo-, and enantio-selectivity [2]. They can catalyze the hydrolysis (and synthesis) of long-chain triglycerides to fatty acids, diacylglycerol, monoacylglycerol, and glycerol known as carboxylesterases [3, 4]. Besides hydrolysis activity, they display interesterification, esterification, aminolysis, and alcoholysis activities, which are contributing to a wide range of industries [5, 6].

Therefore, lipases have been used in biotechnology industries to perform catalytic reactions including transesterification, esterification, and interesterification [7, 8]. Lipases are also used for industrial purposes, in detergent industry as additive in washing powder [9], textile industry to enhance absorbency of fabric [10], the manufacture of biodegradable polymers or compounds [11], and various transesterification reactions [12].

The microbial lipases are more valuable compared to those derived from plants or animals due to their variety of catalytic activities available, high yield production, and simplicity of genetic manipulation, absence of seasonal fluctuations, regular supply, more stability, and more safety and convenience, and the growth rate of microorganisms is very high in economical media [13, 14].

However, microbes like bacteria, fungus, and yeast are found to be foremost sources for lipase synthesis. The microbial lipases can be synthesized in shorter time with high quality and they are a good alternative to reduce the cost of lipase production [15]. However, the optimal growth conditions should be examined to obtain maximum yield of lipase [16].

Currently bacterial lipases are of great demand because of their potential industrial applications. The bacterial lipase is used as a key enzyme in various industrial applications due to its environmental safety, nontoxic nature, and generation of no hazardous residues [12]. Lipases in food and dairy industries are widely applicable for milk fat hydrolysis, ripening and flavour enhancement in cheese product, and lipolysis of cream and butter fat [7, 17]. *Kocuria polaris* WRS3 lipase production confirmed it to be a viable alkaline lipase option for industrial applications such as leather, fine chemicals, and detergents [18].

Immobilization of lipase is to be done to increase its operational stability and activity in order to employ them more efficiently and economically in both nonaqueous and aqueous solvents [19, 20]. There are three main methods for immobilizing a biological compound: adsorption/electrostatic interaction, entrapment, and covalent attachment. Considering the inherent complex nature of the protein structure, lipase is immobilized using entrapment strategy. It has also been reported that the immobilized lipase could be used four times without significant decrease of activity. Previously, the potential of a *Kocuria* sp. for the production of saline-alkaline lipase enzyme immobilized with magnetic Fe_3_O_4_ nanoparticles for commercial applications was reported [18].

The protein purification when functioning with enzymes is a very complex process since it provides the biological material required for structural, functional, and kinetic research. Using successive fractionation, concentration, and chromatography procedures, the extracellular microbial enzyme may be extracted from the cell-free fermentation broth [7, 21, 22].

The goal of this study was purification, optimization, and immobilization of lipase obtained from *K*. *flava* Y4, a plant probiotic, which was newly isolated during our previous study. Moreover, the spatial geometry of *K*. *flava* lipase active site residues for optimal activity has also been investigated.

## 2. Materials and Method

### 2.1. Microorganism and Extraction Process

In the current investigation, *Kocuria flava* Y4 (GenBank accession no. MT773277), which was locally isolated during our previous study, was used. This strain was maintained on nutrient agar slants at 35°C and stored at 4°C. The nutrient broth medium was inoculated with initial optical density (OD) 0.141 of *K*. *flava* Y4 and the overnight grown culture was then centrifuged at 10,000 rpm for 15 min to separate the pellet and supernatant. For further analysis, the supernatant was stored at 4°C [23].

### 2.2. Enzymes Assay

#### 2.2.1. Protease Assay

The casein method of Kunitz [24], as modified by Walter [25], was used to quantify total protease activity of *K*. *flava* Y4 by using casein (1%) in 50 mM Tris/HC1 buffer, pH 9 or Universal Buffer [26]. Under assay conditions, one unit of enzyme is defined as the amount of enzyme that liberates peptide fragments equal to 1 mg of BSA.

#### 2.2.2. Amylase Assay

The DNSA (3,5-dinitrosalicylic acid) method [27] with some modification was used to carry out amylase assay. The assay mixture consists of 500 *μ*l of 0.1 M phosphate buffer, 500 *μ*l of soluble starch (1% w/v), and properly diluted enzyme solutions having pH 7. The reaction was carried out for 15 min at 50°C. With the addition of 1 mL of 3,5-dinitrosalicylic acid reagent and 2 mL of distilled water, the reaction was stopped. The mixture was quickly chilled in ice water after boiling for 5 mins. The absorbance was measured at 540 nm using a spectrophotometer (Thermo Fisher, India). Under assay conditions, one unit of enzyme activity equaled the quantity of enzyme necessary to catalyze the liberation of reducing sugar equivalent to one mol of D-glucose per minute.

#### 2.2.3. Lipase Assay

By using p-nitrophenyl acetate (p-NPA) as substrate, the lipase activity of *K*. *flava* Y4 in the supernatant was performed. The reaction mixture contains 0.8 mL of 0.1 M sodium phosphate buffer (pH 7), 0.1 mL of culture supernatant, and 0.1 mL of 0.01 M p-NPA dissolved in isopropyl alcohol and was incubated for 30 min at 30 ± 1°C and the reaction was stopped by adding 0.25 mL of 0.1 M sodium carbonate. Using a spectrophotometer, the absorbance of reaction mixture was measured at 410 nm [28]. The amount of enzyme necessary to liberate 1 *μ*mol of p-nitrophenol per minute from p-NPA was defined as one lipase unit. Lowry et al.'s method was applied to calculate total protein concentration [29].

### 2.3. Optimization of Lipase Production

#### 2.3.1. Effect of Supplementary Carbon Sources

Different carbon sources (1% w/w) such as glucose, dextrose, sucrose, maltose, mannose, and fructose were used to estimate the effect of carbon supplements on the synthesis of lipase from *K*. *flava* Y4. The best carbon supplement was used for further studies.

#### 2.3.2. Effect of Supplementary Nitrogen Sources

To determine the optimal nitrogen supplement for lipase production, several nitrogen sources such as yeast extract, peptone, urea, sodium nitrate, and ammonium sulphate were added to the medium at 1% w/w individually. Further studies were conducted with the supplement that proved to be the most effective.

#### 2.3.3. Effect of pH

The effect of pH on lipase production was investigated by incubating *K*. *flava* Y4 at 35°C for 1 hour at various pH levels ranging from 3 to 11. As previously mentioned, p-NPA was used as substrate to determine lipase production spectrophotometrically.

#### 2.3.4. Effect of Temperature

Optimum temperature for lipase production was determined by cultivating *K*. *flava* Y4 at different temperatures ranging from 25°C to 45°C. p-NPA was used as substrate to determine lipase activity spectrophotometrically as per earlier description.

#### 2.3.5. Effect of Agitation Speed

20 mL of *K*. *flava* Y4 broth was exposed to different agitation speeds (100, 110, 120, and 130 rpm) under the incubation temperature of 35°C for 24 h. After completing the incubation period, samples were taken for lipase assay as previously described using p-NPA as the substrate.

#### 2.3.6. Effect of Organic Solvents and Metal Ions on Lipase

After preincubating the enzyme (0.2 ml) for 1 h at 30°C with 0.5 ml of methanol, ethanol, 2-propanol, and *n*-hexane, the influence of organic solvents on enzyme activity was examined using spectrophotometric test. The incubation mixture (0.05 ml) was assayed spectrophotometrically to determine the residual activity. The effect of metal ions on activity after preincubation with 1 mM of MnCl_2_, HgCl_2_, MgCl_2_, KCl, AgCl_2_, and CaCl_2_ in 0.05 M phosphate buffer (pH 6.5) at 30°C for 1 h was examined using a spectrophotometric assay.

#### 2.3.7. Effect of Detergents on Lipase

The incubation of a lipase aliquot (0.05 ml) for 1 h at 30°C in 0.05 M phosphate buffer (pH 6.5) having 1% (v/v) detergents (Tween 80, *β*-mercaptoethanol, and SDS (SRL)) was carried out to investigate the effect of different detergents on lipase activity. After the incubation period, the activity was evaluated using a spectrophotometric analysis.

### 2.4. Determination of Half-Life Period

Thermal stability along with half-life time of the lipase was observed by incubating it for 60 min at temperatures ranging from 40 to 80°C. The activity of enzymes was measured at regular intervals.

Lipase half-life (*t*_1/2_) [30] was determined using the following equations:

(1) $$\begin{array}{c} \ln\;A_{t}=\ln\;A_{0}-k_{d}\cdot t, \end{array}$$

(2) $$\begin{array}{c} t_{1/2}=\frac{\ln\;0.5}{-k_{d}}, \end{array}$$

where *A*_0_ is the initial activity and *A*_*t*_ is the activity after heat treatment for *t*_min_.

### 2.5. Determination of *K*_*m*_ and *V*_max_

Olive oil with various concentrations ranging from 0.5% to 2% (w/v) (pH 7.0) was used to measure the initial reaction rate of lipase. For each substrate concentration, enzyme activity per unit time was evaluated after 24 min of incubation at 37°C. The Lineweaver–Burk plot was used to obtain the values of *K*_*m*_ and *V*_max_ [23].

### 2.6. Immobilization Studies

The overnight grown culture was added at 1 : 1 ratio with sodium alginate solution (4%) and eluted drop by drop using a syringe into 0.25 M CaCl_2_.2H_2_O solution, which was left for 2 h to get immobilized beads of cells having a diameter of 2 mm. Sterile distilled water was used to rinse the synthesized alginate beads, which were further used for enzyme assay [31]. The quantity of lipase that produced 1 mole of pNPA under assay condition was determined as one unit of enzyme. According to Dey et al. [31], differences between free and immobilized enzyme activity were measured to determine the immobilization ability.

### 2.7. Purification of Protein

The optimized growth condition was maintained for overnight grown culture and it was centrifuged at 15,000 rpm for 20 minutes at 4°C after 24 h incubation at 37°C. The obtained supernatant was precipitated with 10–70% ammonium sulphate fractionation to yield an enzyme precipitate. The collected precipitates were dissolved in 5 mL of phosphate buffer (pH 7.2) and kept overnight for dialysis against 500 mL of the same buffer. The dialyzed solution was passed over an anion-exchange DEAE-Sepharose Fast Flow column chromatography (GE Healthcare) and Sepharose-6B gel filtration chromatography kit (GeNei) at a flow rate of 1 mL/min. Elution was initially achieved by the equilibrium buffer. The enzyme activity of each fraction was tested. As per Lowry et al.'s method [23], Bovine serum albumin (BSA) was used as the standard to assess lipase activity at each stage of enzyme purification.

### 2.8. Molecular Mass Determination by SDS-PAGE

According to Sundararajan et al., 4% (w/v) stacking gel and 10% (w/v) separating gel were used to perform sodium dodecyl sulphate-polyacrylamide gel electrophoresis (SDS-PAGE) for the determination of molecular weight and purity of the lipase [32]. Gel was stained with Coomassie Brilliant Blue R250 after electrophoresis. The standard protein molecular weight marker (HiMedia, India) was used to determine the molecular weight of the lipase.

## 3. Results

### 3.1. Enzyme Activity

Lipase activity of *K*. *flava* Y4 was found to be 41 ± 0.64 U/mL, which was assessed by the p-NPA assay. Maximum amylase activity and protease activity were found to be 32.7 ± 0.87 U/mL and 5 ± 0.32 U/mL, respectively (Table 1). This study indicated that *K*. *flava* Y4 had maximum lipase activity in comparison to other two enzymes (amylase and protease); therefore, subsequent experiments were carried out with lipase.

### 3.2. Optimization of Lipase Production

#### 3.2.1. Effect of Supplementary Carbon Sources

**Figure 1:**
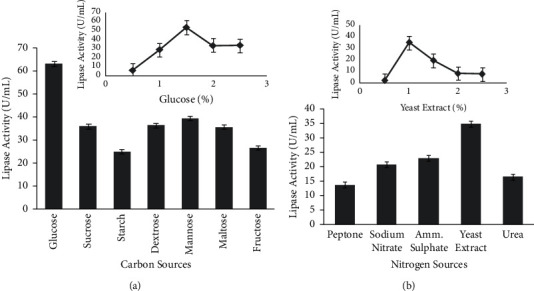
Effect of different (a) carbon sources and (b) nitrogen sources on lipase production by *K*. *flava* Y4.

Single factor analysis was used for optimization of media components. The medium containing 1% glucose as carbon source showed highest lipase activity of 63.33 ± 1.18 U/mL followed by mannose, dextrose, sucrose, starch, maltose, and fructose showing lipase activity of 38.93 ± 0.33, 36.53 ± 0.288, 36.16 ± 1.014, 25.3 ± 0.45, 36.033 ± 0.47, and 26.86 ± 0.38 U/mL, respectively. Among various concentrations of glucose supplements ranging from 0.5 to 2.5% to the medium, 1.5% of glucose concentration showed highest lipase activity (Figure 1(a)).

#### 3.2.2. Effect of Supplementary Nitrogen Sources

Among different nitrogen sources such as urea, peptone, yeast extract, ammonium sulphate, and sodium nitrate in the medium, the highest lipase activity (34.86 ± 0.38 U/mL) was observed in the case of 1% yeast extract, followed by 1% each of peptone (13.7 ± 0.287 U/mL), ammonium sulphate (23.06 ± 0.438 U/mL), urea (16.53 ± 0.28 U/mL), and NaNO_3_ (20.76 ± 0.23 U/mL). Further, various concentrations of yeast extract ranging from 0.5 to 2.5% were supplemented to the medium and the highest lipase activity was observed in 1% concentration (Figure 1(b)).

#### 3.2.3. Effect of Physicochemical Parameters

**Figure 2:**
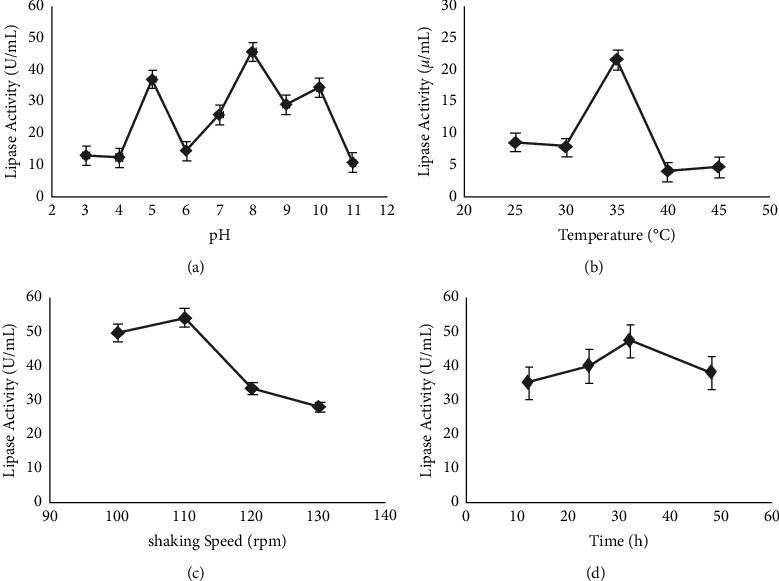
Effects of initial pH (a), temperature (b), and shaking speed (c) on lipase production by *K*. *flava* Y4.

The optimum pH was determined by assessing the lipase activity in the cell-free *K*. *flava* Y4 at different pH (3–11). The pH ranges from 7 to 11 showed maximum lipase activity; however, the highest lipase activity (45.96 ± 0.47 U/mL) was observed at pH 8 (Figure 2(a)). This shows that *K*. *flava* Y4 strain is active over a broad range of pH.

Optimum temperature has a key role in enzyme activity. The lipase activity at different temperatures, namely, 25, 30, 35, 40, and 45°C, was found to be 8.55 ± 0.18 U/mL, 7.8 ± 0.33 U/mL, 21.63 ± 0.29 U/mL, 4 ± 0.47 U/mL, and 4.7 ± 0.205 U/mL, respectively, thereby demonstrating the highest lipase activity (21.63 ± 0.29 U/mL) at 35°C (Figure 2(b)).

Optimum shaking speed plays a vital role in the production of enzymes. The lipase activity at different shaking speed, namely, 100, 110, 120, and 130 rpm, was found to be 33.63 ± 0.55 U/mL, 54.33 ± 0.53 U/mL, 28.03 ± 0.44 U/mL, and 50 ± 0.47 U/mL, respectively, thereby demonstrating the highest lipase activity (54.33 ± 0.53 U/mL) at 110 rpm (Figure 2(c)).

The lipase activity at different incubation time, namely, 12, 24, 32, and 48 h, was found to be 35.1 ± 0.51 U/mL, 40.06 ± 0.47 U/mL, 47.33 ± 0.56 U/mL, and 38.133 ± 0.47 U/mL, respectively, hence demonstrating the highest lipase activity (47.33 ± 0.56 U/mL) at 32 h (Figure 2(d)).

#### 3.2.4. Effect of Organic Solvents and Metal Ions on Lipase

The sensitivity of lipases to solvents varies, although it is generally agreed that polar water-miscible solvents are more destabilizing than water-immiscible solvents. The lipase from *K*. *flava* Y4 demonstrated relative activity of 103.21 ± 0.825% depicting highest stability on exposure to 30°C for 1 h. In comparison to the control, addition of 70% isopropanol to the lipase mixture resulted in a 3% instantaneous increase in lipolytic activity, whereas the extracellular lipase from *K*. *flava* Y4 in the presence of other organic solvents like 70% methanol, 70% ethanol, and 70% *n*-hexane showed relative activity of 57.3 ± 0.73%, 55.18 ± 1.57%, and 53.7 ± 0.73%, respectively, showing inhibition of lipase activity (Table 2).

The effect of different metal ions on the activity of the lipase is shown in Table 3. All metal ions had an effect on lipase activity, although Mn^2+^ had the strongest inhibitory activity (21.87 ± 1.33%) followed by MgCl_2_ (41.51 ± 1.01%), HgCl_2_ (42.13 ± 1.53%), KCl (55.53 ± 1.4%), CaCl_2_ (56.74 ± 1.2%), and AgCl_2_ (62.59 ± 1.16%).

#### 3.2.5. Effect of Detergents on Lipase

Table 4 shows the effects of detergents on lipase activity. After 30 mins at 60°C, pH 6.5, the addition of 1% *β*-mercaptoethanol to the lipase mixture totally reduced the enzyme activity; however, it was also partially inhibited in the presence of 1% SDS (96.18 ± 1%) and Tween 80 (71.76 ± 1.059%).

Moreover, the lipase activity of standard bacteria (*Bacillus subtilis* (ATCC 6633)) was 33.27 ± 0.61 U/mL, which was reduced dramatically 20-fold compared to *Kocuria flava* Y4 lipase activity of 54.33 ± 0.53 U/mL (Table 5). This indicates a greater enzyme activity of *K*. *flava* Y4.

### 3.3. Thermal Stability Test

**Figure 3:**
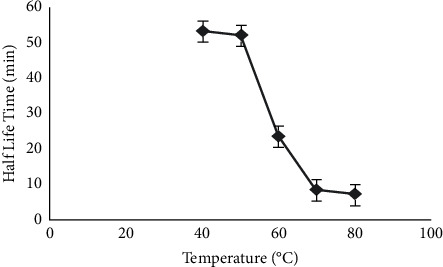
Thermal stability study.

The thermal stability of the enzyme was studied from 40 to 80°C. The enzyme stability was observed even at higher temperature, that is, 80°C and lipase half-life was found to be 53.72 min at 40°C, 52.5 min at 50°C, 23.84 min at 60°C, 8.41 min at 70°C, and 7.04 min at 80°C (Figure 3).

### 3.4. Determination of *K*_*m*_ and *V*_max_

**Figure 4:**
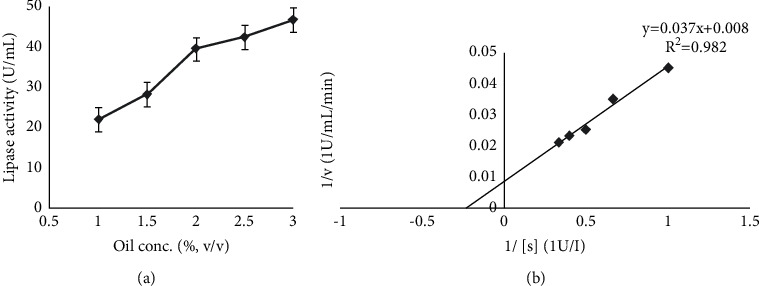
(a) Effect of olive oil concentration on lipase activity. (b) Determination of *K*_*m*_ and *V*_max_.

The initial rate of hydrolysis varied linearly with substrate concentration.  Figure 4(a) shows that maximum lipase activity (39.6 U/mL) was obtained at substrate (olive oil) concentration of 2% w/v for *K*. *flava* Y4. However, the lipase activity decreased to 23.5 U/mL at 1.5% w/v olive oil concentration and the activity was found to be lowest (17.1 U/mL) at 1% w/v olive oil concentration.

The Lineweaver–Burk plot is the most popular method, with the benefit of plotting the variables v and [S] on distinct axes.  Figure 4(b) shows the reaction speeds, v, at various olive oil concentrations, [S], and all the v and [S] rearrangement values, namely, 1/v, 1/[S], v/[S], and [S]/v. The values of *V*_max_ and *K*_*m*_ were determined by using the Lineweaver–Burk plot. The *K*_*m*_ and V_max_ values were found to be 4.625 U/mL and 125 U/mL/min, respectively.

### 3.5. Immobilization Study

**Figure 5:**
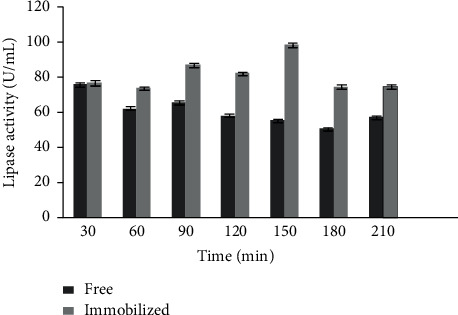
Comparison between free and immobilized lipase activity obtained from *K*. *flava* Y4.

The catalytic activity of lipase obtained from immobilized and free *K*. *flava* Y4 were compared (Figure 5). According to Casas-Godoy et al., the significant discrepancies in lipase catalytic activity were most likely related to enzyme immobilization [33]. Adsorption of lipase has been shown to impact catalytic activity by altering enzyme structure and increasing molecular rigidity [34]. As observed in Figure 5, catalytic activity of lipase obtained from free *K*. *flava* Y4 was highest at 30 min (75.66 ± 1.18 U/mL) followed by the lipase activity at 60 min (62.23 ± 0.85 U/mL), 90 min (65.56 ± 1.08 U/mL), 120 min (58.2 ± 0.99 U/mL), 150 min (55.36 ± 1 U/mL), 180 min (50.53 ± 0.92 U/mL), and 210 min (57.2 ± 0.94 U/mL). However, the catalytic activity of lipase obtained from immobilized *K*. *flava* Y4 increased to ∼2-fold (98.63 ± 1.17 U/mL) when compared to the catalytic activity of lipase obtained from free *K*. *flava* Y4 (55.36 ± 1 U/mL) at 150 min. Catalytic activity of lipase obtained from immobilized *K*. *flava* was found to be highest at 98.63 ± 1.17 U/mL (150 min) followed by 87.13 ± 0.94 U/mL (90 min), 82.26 ± 0.9 U/mL (120 min), 77 ± 1.47 U/mL (30 min), 74.66 ± 1.13 U/mL (210 min), 74.6 ± 1.17 U/mL(180 min), and 73.86 ± 1.01 U/mL(60 min). It has also been reported that lipase obtained from immobilized *K*. *flava* Y4 could be used four times without significant decrease of activity.

### 3.6. Purification of Lipase

The summary of the lipase purification is shown in Table 5. Using the lipase activity assay described earlier, the supernatant had a hydrolytic activity against p-NPA of 0.23 U/mL. The ammonium sulphate precipitation of the cell-free extract was carried out at a range of 10–70% saturation. Salt precipitation with ammonium sulphate at saturation point of 0–60% resulted in precipitates. The enzyme activity in these fractions was not completely separable from the supernatant and was therefore not characterized. 70% fraction did not show any activity. The 30–60% fraction showed the maximum specific activity, which was subsequently used for further purification. After sequential application of salt precipitation and DEAE-Sepharose fast-flow column chromatography, the lipase was finally purified to ∼15-fold with a yield of 96 U/mL (Table 6).

### 3.7. SDS-PAGE Analysis

**Figure 6:**
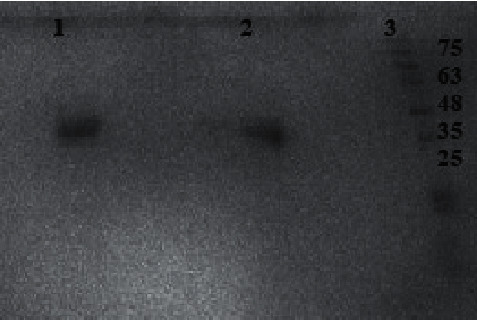
SDS-PAGE illustrates the presence of purified lipase enzyme. Lane 1 shows the ammonium sulphate precipitated, lane 2 illustrates the lipase obtained after purification, and lane 3 lipase shows the position of molecular markers (kDa).

The molecular mass of *K*. *flava* Y4 lipase was determined using SDS-PAGE and was found to be around 45 kDa (Figure 6).

## 4. Discussion

Researches conducted earlier have delineated the lipase activity of *Kocuria flava* ASU5 to be 16.04 ± 0.43 U/mL [33]. In addition to that, some other studies demonstrated the role of glucose in enhancing lipase activity. Increase in glucose concentration ranging from 1.5 to 3.5% increases the lipase activity; however, 2.5% glucose concentration exhibits the maximum lipase activity [34]. The enhanced lipase activity was also observed in the medium supplemented with yeast extract [35]. Both inorganic and organic nitrogen sources provide nitrogen, amino acids, and cell growth factors required for enzyme synthesis [36]. pH also plays a key role in lipase production. Maximum lipase production by the bacterial strain was achieved at pH 6–7 [36], whereas a reduced enzyme activity was observed under acidic conditions. Yet another study demonstrated maximum lipase activity by the bacteria at pH higher than 7 [7]. In a similar study, it was reported that lower temperatures enhanced lipase activities, and a little rise in temperature up to 38°C can enhance the production of lipase [37–39]; however, temperatures above 40°C substantially decreased the lipase activities [40]. Lipase from *Candida rugosa* had a half-life of 22.8 min at 55°C [30]. Further, longer incubation periods resulted in lower lipase activity, which could be related to nutrition reduction, change in pH of the medium, toxic end products accumulation, and moisture loss [41]. Brozzoli et al. revealed that shaking speed had a substantial impact on lipase synthesis by *Candida cylindracea* NRRL Y-17506 in olive mill wastewater [42]. Changes in agitation speed of *Rhizopus arrhizus* cultures affects both rate and amount of cell growth and production of lipase in shake flasks due to change in oxygen transfer rate [43].

Our results were confirmed by similar results shown by BTL-1 and BTL-2 lipases [44, 45] with enhanced activity in the presence of 30% methanol and 30% ethanol, respectively. A small coating of water molecules is thought to remain tightly linked to the enzyme, acting as a protective sheath along the enzyme's hydrophilic surfaces and allowing the enzyme to maintain its original shape [46]. The lipase's stability in organic solvents indicates probable applications in nonaqueous biocatalysis. The action of metal ions has been hypothesized to be due to a change in the solubility and behavior of ionized fatty acids at interfaces, as well as a change in the catalytic characteristics of the enzyme [47]. Hg^2+^ showed a strong inhibitory effect on lipase activity, implying that it alters enzyme structure, as has been studied for other thermophilic lipases [48–50]. Schmidt-Dannert et al. [44] reported a complete loss of lipolytic activity in the presence of Tween 80, which is consistent with our findings. Nawani et al. [46] also reported a complete decrease of activity in the presence of SDS. It was also studied that the immobilized lipase may be utilized four times without losing its activity. The potential of a *Kocuria* sp. for the commercial production of saline-alkaline lipase enzyme bound with magnetic Fe_3_O_4_ nanoparticles was previously investigated [18].

Our finding is validated by a similar finding that reported maximum lipase activity of *Candida rugosa* with 0.5% w/v olive oil [51]. Amidst natural oils, olive oil has been emerged as one of the best inductors and substrates for lipase synthesis [52]. Hydrolysis rate of coconut oil, tallow, and olive oil by lipase produced from *Candida rugosa* changed linearly with oil concentration [53]. In a previous study, a Lineweaver–Burk plot provided *K*_*m*_ (7.72 mM), *V*_max_ (90.91 U/mL/min) values for *Bacillus thermoamylovorans* BHK67 lipase (BTL) [54].

Previously, the lipase enzyme has been purified and/or manipulated by several researchers. The *P*. *aeruginosa* IGB 83 lipase has been reported to be purified by a combination of ammonium sulphate precipitation and gel exclusion but with lower yield (16.4%) [24]. Ogino et al. [55] reported the purification of *P*. *aeruginosa* LST-03 lipase by ion exchange and hydrophobic interaction chromatography achieving 34.7-fold purification and 12.6% yield. In addition to that, the potential of a *Kocuria* sp. for the production of saline-alkaline lipase enzyme immobilized with magnetic Fe_3_O_4_ nanoparticles for commercial applications was reported [18]. The molecular weight of the lipases from *Kocuria* sp. has been reported to be between 19 and 60 kDa [56].

## 5. Conclusion

In the current study, different physicochemical factors such as pH, temperature, shaking speed, and incubation duration were used to modulate lipase activity of *Kocuria flava* Y4 (isolated during our earlier investigation). For the first time, *K*. *flava* Y4 lipase was purified using a combination of ammonium sulphate precipitation, dialysis, DEAE-Sepharose anion exchange chromatography, and Sepharose-6B gel filtration chromatography, yielding ∼15-fold purified lipase with a final yield of 96 U/mL. At a temperature of 35°C and a pH of 8, the purified lipase showed maximum hydrolytic activity. In the presence of glucose and yeast extract as carbon and nitrogen sources, respectively, an increase in the enzyme activity was observed. Critical analysis of lipase through SDS-PAGE showed interesting ﬁndings. It has a molecular weight of 45 kDa and optimal activity at 40°C and pH 8.0. This indicated that lipases from *K*. *flava* Y4 has signiﬁcant potential for commercialization as a biocatalyst for industrial purposes. In comparison with other commercially available enzymes, it is believed that engineering present lipase will allow attainment of enzymes with new remarkable characteristics for a speciﬁc application.

## Figures and Tables

**Table 1:** Extracellular enzymes secreted by *K*. *flava* Y4 and their activities.

Enzyme	Enzyme activity (U/ml)
Lipase	41 ± 0.64
Amylase	32.7 ± 0.87
Protease	5 ± 0.32

**Table 2:** Stability of *K*. *flava* Y4 lipase in organic solvents.

Compound (1 mM)	Relative activity (%)
Control	100 ± 3.36
Methanol	57.3 ± 0.73
*n*-Hexane	53.7 ± 0.73
Isopropanol	103.21 ± 0.825
Ethanol	55.18 ± 1.57

**Table 3:** Effect of metal ions on *K*. *flava* Y4 lipase activity.

Compound (1 mM)	Relative activity (%)
Control	100 ± 3.36
MnCl_2_	21.87 ± 1.33
HgCl_2_	42.13 ± 1.53
AgCl_2_	62.59 ± 1.16
CaCl_2_	56.74 ± 1.2
KCl	55.53 ± 1.4
MgCl_2_	41.51 ± 1.01

**Table 4:** Effect of detergents and enzyme inhibitors on *K*. *flava* Y4 lipase activity.

Compound (1 mM)	Relative activity (%)
Control	100 ± 2.55
SDS	96.18 ± 1
Tween 80	71.76 ± 1.059
*β*-Mercaptoethanol	0

**Table 5:** Comparison of enzyme activity of *K*. *flava* Y4 lipase with bacterial lipase *Bacillus subtilis* (ATCC 6633) at optimum pH (45.96 ± 0.47 U/mL), temperature (23.066 ± 0.09 U/mL), and shaking speed (54.33 ± 0.53 U/mL).

Organisms	Enzyme activity (U/ml)
*Kocuria flava* Y4	54.33 ± 0.53 U/mL
Bacteria (*Bacillus subtilis* (ATCC 6633))	33.27 ± 0.61 U/mL

**Table 6:** Flow sheet of the *K*. *flava* Y4 lipase purification.

Enzyme	Volume (*μ*l)	Units of lipase (U/mL)	Specific activity (U/mg)	Purification fold
Crude	20,000	22	3.6	—
Partially purified	2000	36	36	10
Purified	200	96	533.3	14.81

## Data Availability

The data used to support the findings of this study are included within the article.

## References

[1] Kirk O., Borchert T. V., Fuglsang C. C. (2002). Industrial enzyme applications. *Current Opinion in Biotechnology*.

[2] Jaeger K. E., Dijkstra B. W., Reetz M. T. (1999). Bacterial biocatalysts: molecular biology, three-dimensional structures, and biotechnological applications of lipases. *Annual Review of Microbiology*.

[3] Gupta R., Gupta N., Rathi P. (2004). Bacterial lipases: an overview of production, purification and biochemical properties. *Applied Microbiology and Biotechnology*.

[4] Kapoor M., Gupta M. N. (2012). Lipase promiscuity and its biochemical applications. *Process Biochemistry*.

[5] Fujii T., Tatara T., Minagawa M. (1986). Studies on applications of lipolytic enzyme in detergency I. Effect of lipase from candida cylindracea on removal of olive oil from cotton fabric. *Journal of the American Oil Chemists’ Society*.

[6] Sharma R., Chisti Y., Banerjee U. C. (2001). Production, purification, characterization, and applications of lipases. *Biotechnology Advances*.

[7] Linko Y. Y., Lämsä M., Wu X., Uosukainen E., Seppälä J., Linko P. (1998). Biodegradable products by lipase biocatalysis. *Journal of Biotechnology*.

[8] Hasan F., Aamer A. S., Shah A. A., Hameed A. (2006). Industrial applications of microbial lipases. *Enzyme and Microbial Technology*.

[9] Treichel H., de Oliveira D., Mazutti M. A., Di Luccio M., Oliveira J. V. (2010). A review on microbial lipases production. *Food and Bioprocess Technology*.

[10] Linfield W. M., Barauskas R. A., Sivieri L., Serota S., Stevenson R. W. (1984). Enzymatic fat hydrolysis and synthesis. *Journal of the American Oil Chemists’ Society*.

[11] Falch E. A. (1991). Industrial enzymes—developments in production and application. *Biotechnology Advances*.

[12] Arafa R. (2017). Immobilization and characterization of lipase loaded on Fe_3_O_4_ nanoparticles and produced from haloalkalophilis *Kocuria Polaris*-WRS3. *International Journal of Information Research and Review*.

[13] Moraleda-Muñoz A., Shimkets L. J. (2007). Lipolytic enzymes in *Myxococcus xanthus*. *Journal of Bacteriology*.

[14] Javed S., Azeem F., Hussain S. (2018). Bacterial lipases: a review on purification and characterization. *Progress in Biophysics and Molecular Biology*.

[15] Rajendran A., Palanisamy A., Thangavelu V. (2009). Lipase catalyzed ester synthesis for food processing industries. *Brazilian Archives of Biology and Technology*.

[16] Karadzic I., Masui A., Zivkovic L. I., Fujiwara N. (2006). Purification and characterization of an alkaline lipase from *Pseudomonas aeruginosa* isolated from putrid mineral cutting oil as component of metalworking fluid. *Journal of Bioscience and Bioengineering*.

[17] Reetz M. T. (2013). Biocatalysis in organic chemistry and biotechnology: past, present, and future. *Journal of the American Chemical Society*.

[18] Mendes A. A., Oliveira P. C., de Castro H. F. (2012). Properties and biotechnological applications of porcine pancreatic lipase. *Journal of Molecular Catalysis B: Enzymatic*.

[19] Villeneuve P., Muderhaw J. M., Graille J., Haas M. J. J. (2000). Lipase-catalyzed esterification of Betulinic acid using phthalic anhydrid in organic solvent media: study of reaction parameters. *Molecular Catalysis B Enzyme*.

[20] Mostafa H., El-Hadi A. A. (2010). Immobilization of *Mucor racemosus* NRRL 3631 lipase with different polymer carriers produced by radiation polymerization. *Malaysian Journal of Microbiology*.

[21] Kademi A., Aït-Abdelkader N., Fakhreddine L., Baratti J. (2000). Purification and characterization of a thermostable esterase from the moderate thermophile *Bacillus circulans*. *Applied Microbiology and Biotechnology*.

[22] Sharma A., Bhattacharyya K. G. (2005). *Azadirachta indica* (neem) leaf powder as a biosorbent for removal of Cd(II) from aqueous medium. *Journal of Hazardous Materials*.

[23] Negi S., Banerjee R. (2009). Characterization of amylase and protease produced by *Aspergillus awamori* in a single bioreactor. *Food Research International*.

[24] Kunitz M. (1947). Crystalline soybean trypsin inhibitor. *Journal of General Physiology*.

[25] Walter H. (1984). Proteases and their inhibitors. 2. 15. 2 Method with haemoglobin, casein and azocoll as substrate. *Methods of Enzymatic Analysis*.

[26] Stauffer C. E. (1989). Viscometric measurements of enzyme activity. *Enzyme Assays for Food Scientists*.

[27] Miller G. L. (1959). Use of dinitrosalicylic acid reagent for determination of reducing sugar. *Analytical Chemistry*.

[28] Khosla K., Rathour R., Maurya R. (2017). Biodiesel production from lipid of carbon dioxide sequestrating bacterium and lipase of psychrotolerant *Pseudomonas* sp. ISTPL3 immobilized on biochar. *Bioresource Technology*.

[29] Lowry O. H., Nira J., Rosebrough A. L., Randall R. J. (1951). Protein measurement with the Folin phenol reagent. *Journal of Biological Chemistry*.

[30] Miranda M., Urioste D., Livia T., Andrade S., Mendes A. A., de Castro H. F. (2011). Assessment of the morphological, biochemical, and kinetic properties for *Candida rugosa* lipase immobilized on hydrous niobium oxide to be used in the biodiesel synthesis. *Enzyme Research*.

[31] Dey G., Bhupinder S., Banerjee R. (2003). Immobilization of alpha-amylase produced by *Bacillus circulans* GRS 313. *Brazilian Archives of Biology and Technology*.

[32] Sundararajan S., Kannan C. N., Chittibabu S. (2011). Alkaline protease from *Bacillus cereus* VITSN04: potential application as a dehairing agent. *Journal of Bioscience and Bioengineering*.

[33] Casas-Godoy L., Marty A., Sandoval G., Ferreira-Dias S. (2013). Optimization of medium chain length fatty acid incorporation into olive oil catalyzed by immobilized Lip2 from *Yarrowia lipolytica*. *Biochemical Engineering Journal*.

[34] Xie W., Ma N. (2010). Enzymatic transesterification of soybean oil by using immobilized lipase on magnetic nano-particles. *Biomass and Bioenergy*.

[35] Khorami M., Maryam H. Y., Ghasem N. (2012). Lipase production in solid state fermentation using *Aspergillus niger*: response surface methodology. *International Journal of Engineering*.

[36] Larbidaouadi K., Benattouche Z., Abbouni B. (2015). Screening selection identification production and optimization of bacterial lipase isolated from industrial rejection of gas station. *International Journal of Biotechnology Allied Fields*.

[37] Zhang Y., Cheng F., Wang Y. (2005). Study of elemental concentration and distribution in human femoral head by PIXE and SRXRF. *International Journal of PIXE*.

[38] Gaur R., Gupta A., Khare S. K. (2008). Purification and characterization of lipase from solvent tolerant *Pseudomonas aeruginosa* PseA. *Process Biochemistry*.

[39] Yuan D., Lan D., Xin R., Yang B., Wang Y. (2016). Screening and characterization of a thermostable lipase from marineStreptomycessp. strain W007. *Biotechnology and Applied Biochemistry*.

[40] Cao J., Dang G., Li H. (2015). Identification and characterization of lipase activity and immunogenicity of LipL from *Mycobacterium tuberculosis*. *PLoS One*.

[41] Imandi S. B., Karanam S. K., Garapati H. R. (2013). Use of plackett-burman design for rapid screening of nitrogen and carbon sources for the production of lipase in solid state fermentation by *Yarrowia lipolytica* from mustard oil cake (*Brassica napus*). *Brazilian Journal of Microbiology*.

[42] Brozzoli V., Crognale S., Sampedro I., Federici F., D’Annibale A., Petruccioli M. (2009). Assessment of olive-mill wastewater as a growth medium for lipase production by *Candida cylindracea* in bench-top reactor. *Bioresource Technology*.

[43] Elibol M., Ozer D. (2000). Influence of oxygen transfer on lipase production by *Rhizopus arrhizus*. *Process Biochemistry*.

[44] Schmidt-Dannert C., Sztajer H., Stöcklein W., Menge U., Schmid R. D. (1994). Screening, purification and properties of a thermophilic lipase from *Bacillus thermocatenulatus*. *Biochimica et Biophysica Acta (BBA)—Lipids and Lipid Metabolism*.

[45] Schmidt-Dannert C. M., Atomi H., Schmid R. D. (1996). Thermoalkalophilic lipase of *Bacillus thermocatenulatus*. I. Molecular cloning, nucleotide sequence, purification and some properties. *Biochimica et Biophysica Acta (BBA)-Lipids and Lipid metabolism*.

[46] Nawani N., Dosanjh N. S., Kaur J. (1998). A novel thermostable lipase from a thermophilic *Bacillus* sp.: characterization and esterification studies. *Biotechnology Letters*.

[47] Lesuisse E., Schanck K., Colson C. (1993). Purification and preliminary characterization of the extracellular lipase of *Bacillus subtilis* 168, an extremely basic pH-tolerant enzyme. *European Journal of Biochemistry*.

[48] Lee D. W., Koh Y. S., Kim K. J. (1999). Isolation and characterization of a thermophilic lipase from Bacillus thermoleovoransID-1. *FEMS Microbiology Letters*.

[49] Handelsman T., Shoham Y. (1994). Production and characterization of an extracellular thermostable lipase from a thermophilic *Bacillus* sp. *The Journal of General and Applied Microbiology*.

[50] Lee D. W., Kim H. W., Lee K. W., Kim B. C., Choe E. A. (2001). Han-Seung Lee, Doo-Sik Kim, and Yu-Ryang Pyun. Purification and characterization of two distinct thermostable lipases from the gram-positive thermophilic bacterium *Bacillus thermoleovorans* ID-1. *Enzyme and Microbial Technology*.

[51] Abdelmoez W., Mostafa N. A., Mustafa A. (2013). Utilization of oleochemical industry residues as substrates for lipase production for enzymatic sunflower oil hydrolysis. *Journal of Cleaner Production*.

[52] Bornscheuer U. T. (2002). Microbial carboxyl esterases: classification, properties and application in biocatalysis. *FEMS Microbiology Reviews*.

[53] Noor I. M., Hasan M., Ramachandran K. B. (2003). Effect of operating variables on the hydrolysis rate of palm oil by lipase. *Process Biochemistry*.

[54] Sharma A., Meena K. R., Kanwar S. S. (2018). Molecular characterization and bioinformatics studies of a lipase from *Bacillus thermoamylovorans* BHK67. *International Journal of Biological Macromolecules*.

[55] Ogino H., Ishikawa H. (2001). Enzymes which are stable in the presence of organic solvents. *Journal of Bioscience and Bioengineering*.

[56] Karl-Erich K., Reetz M. T. (1998). Microbial lipases form versatile tools for biotechnology. *Trends in Biotechnology*.

